# Obstetric Patients Requiring Intensive Care: A One Year Retrospective Study in a Tertiary Care Institute in India

**DOI:** 10.1155/2014/789450

**Published:** 2014-03-25

**Authors:** Niyaz Ashraf, Sandeep Kumar Mishra, Pankaj Kundra, P. Veena, S. Soundaraghavan, S. Habeebullah

**Affiliations:** ^1^Department of Anaesthesiology and Critical Care, Jawaharlal Institute of Postgraduate Medical Education and Research (JIPMER), Dhanvantri Nagar, Pondicherry 605006, India; ^2^Department of Obstetrics and Gyanaecology, Jawaharlal Institute of Postgraduate Medical Education and Research (JIPMER), Dhanvantri Nagar, Pondicherry 605006, India

## Abstract

*Background and Objectives.* Critically ill obstetric patients are a particularly unique cohort for the intensivist. The objective of this study was to review the indications for admission, demographics, clinical characteristics, and outcomes of obstetric patients admitted to intensive care unit of a medical college hospital in southern India and to identify conditions associated with maternal mortality. * Design.* Retrospective analysis of pregnant/postpartum (up to 6 weeks) admissions over a 1-year result. We studied 55 patients constituting 11.6% of mixed ICU admissions during the study period.* Results.* The mean APACHE (acute physiology and chronic health evaluation) II score of patients at admission was 11.8. Most of the patients (76%) were admitted in the antepartum period. The commonest indications for ICU admission were obstetric haemorrhage (51%) and hypertensive disorders of pregnancy (18%). 85% of patients required mechanical ventilation and 78% required inotropic support. *Conclusions.* Maternal mortality was 13%, and the majority of the deaths were due to disseminated intravascular coagulation and multiorgan failure, following an obstetric haemorrhage. A dedicated obstetric ICU in tertiary hospitals can ensure that there is no delay in patient management and intensive care can be instituted at the earliest.

## 1. Introduction

Obstetric patients are a particularly unique cohort for the intensivist. These patients are young and otherwise healthy; their management is challenged by concerns for fetal viability, altered maternal physiology, and diseases specific to pregnancy. There are several reports on critically ill obstetric patients, but data from India is scarce despite huge number and wide stratum of obstetric population [1, 2]. The present data was collected to understand the elements influencing the maternal outcome and identify preventable factors amongst them that were responsible for adverse maternal/fetal outcome in underdeveloped regions.

The objectives of our study were to review the indications for admission, demographics, clinical characteristics, and outcomes of obstetric patients admitted in our ICU in the last one year and to identify conditions associated with maternal mortality.

## 2. Materials and Methods

The present study was conducted in a 6-bedded multidisciplinary intensive care unit (ICU) at 1500 bedded tertiary care center, Jawaharlal Institute of Postgraduate Medical Education and Research (JIPMER) in Pondicherry, India. Obstetric service of the hospital provides antenatal care for 110,000 out-patients and 20,000 in-patients annually, with neonatologists available round the clock. All parturients and 42-day postpartum patients admitted to the ICU between August 1, 2012, and July 31, 2013, were included in the study. Patients were managed by ICU team, consisting of an Anaesthesiology Consultant, Anaesthesiology residents, and Critical Care fellows. The admitting and referring obstetric unit provided consultation on daily basis.

The data were collected in all obstetric patients (pregnant or within the 6-week postpartum period) admitted to the Anaesthesiology ICU over a period of one year (August 1, 2012, to July 31, 2013). Multidisciplinary ICU is a 6-bed closed unit, which admits nearly 400 patients annually.

The data collected included basic demographic characteristics, obstetric/medical history and diagnosis at admission, APACHE 2 score at time of admission, ICU course and length of stay, and treatment given and outcome. In addition clinical indication that prompted ICU admission (organ failures as defined in Table 1) was also recorded. Disease process identified to be responsible for the patient's critical illness was referred to as the primary diagnosis and was categorized as obstetric or non-obstetric.

## 3. Results

A total of 55 obstetric patients were admitted in ICU from August 1, 2012, to July 31, 2013. This represented 0.38 percent of deliveries during this time. The mean age of patients admitted was 27 ± 4.9 years. Out of 55 patients, 42 were admitted in the ante partum period, 9 were admitted in the postpartum period, and 4 were postabortal. 44% of the patients were transported or referred to JIPMER because of the high-risk status. The baseline characteristics of the patients at admission to our hospital are shown in Table 2.

Most of the patients (*n* = 42, 76%) were admitted in the antepartum period, majority (*n* = 46, 82%) for obstetric reasons, of which postpartum haemorrhage (*n* = 15) was the most common (Table 3). Obstetric haemorrhage and subsequent haemodynamic collapse were the commonest reasons for ICU admission, comprising 51% of all ICU admissions. 11 out of these 28 (39.2%) were admitted with antepartum haemorrhage (placenta praevia, placental abruption, and placenta accreta) and 9 of them were referred from peripheral hospitals ill-equipped for better management in tertiary care hospital. Fifteen patients were admitted with postpartum hemorrhage, of which 10 patients developed hemorrhage during/after caesarean section. Uterine devascularization and hysterectomy was performed in 3 of these patients, as life-saving measure to stop refractory bleeding.

Pregnancy-related hypertensive disorders were the second common obstetric causes of admission (*n* = 11, 18%); Five out of 11 such patients were admitted to the ICU due to pulmonary edema requiring ventilatory support. Two patients were admitted with eclamptic seizures and altered sensorium. One of these patients was subsequently diagnosed as Posterior Reversible Encephalopathy Syndrome after MRI Brain (Table 4). All patients recovered fully and were discharged without any focal deficits. Three patients developed renal impairment and renal replacement therapy was initiated in 1 patient. Postabortal sepsis was the basis of ICU admission in 11% (*n* = 6) patients. Four of them were in haemodynamically unstable and required vasopressor support (Table 4).

Forty-seven patients out of 55 admissions (85%) required mechanical ventilation, which was the commonest intervention recorded in ICU. Mean duration of mechanical ventilation was 1.2 ± 1.4 days. Only 1 patient developed ventilator associated pneumonia. She was treated with culture-sensitive antibiotics and made an uneventful recovery.

Cardiac output, Stroke Volume Variation, and Systemic Vascular Resistance were monitored (Table 4) with the FloTrac Vigileo monitor (Edward Lifesciences) in 32 patients (58%), to guide inotropic and fluid management.

There were 7 maternal deaths (13%) in the study period (Table 5) and majority of the deaths were due to disseminated intravascular coagulation and multiorgan failure, following massive obstetric hemorrhage.

## 4. Discussion

Obstetric admissions represented 11.6% of total admissions (475) to our ICU during the study period. This is considerably greater than the statistics mentioned in other studies from India [1, 2]. JIPMER owing to its status as a tertiary care institute of national importance provides free-of-cost, high quality care to nearly 1.7 million patients a year. These patients come from all parts of South India, and many arrive at our hospital unable to afford care elsewhere. This also has a significant bearing on the condition of the patient on arrival here. 44% of the patients in our study were referred from peripheral health centers ill-equipped to manage obstetric emergencies, in terms of availability of blood components and ventilatory support. The maternal mortality rate of obstetric patients in our study was 13%, which was lesser than that in the other studies from India [1, 2]. Six out of the 7 patients who died were referred to us from other hospitals and had to travel distances of more than 50 kilometers, in the process losing precious time. The mean APACHE 2 score of these 6 patients at admission was 22 (predicted mortality of 29%). Of further significance is the fact that 5 of these patients succumbed to DIC and multiorgan failure secondary to obstetric hemorrhage. A systematic review conducted by the WHO found that postpartum haemorrhage is the leading cause of maternal mortality in Africa and Asia, accounting for up to half of the total number of deaths in these regions [3]. Overall, postpartum haemorrhage accounts for an estimated 25% of maternal mortality worldwide [4]. Moreover, the mortality following obstetric haemorrhage is an index of the degree of accessibility of quality health care.

In the light of our experience, a few measures may help reduce maternal mortality in developing countries. There is a need to recognize that socioeconomic factors and lack of infrastructure can vastly influence maternal death than just failure of clinical management. As noted by the WHO, “There is a story behind every maternal death or life-threatening complication” [5]. Addressing the need for accessibility and comprehensiveness of obstetric care, especially in the less connected regions will go a long way towards reducing the maternal mortality. One important area that needs urgent attention is lack of adequately stocked blood banks in peripheral health centres. As discussed by Cruz found an inverse association between donor blood availability and both maternal mortality ratios and risk of death due to postpartum haemorrhage [6]. In addition, dedicated obstetric ICU in tertiary hospitals can ensure there is no delay in patient management and intensive care can be instituted at the earliest.

## Figures and Tables

**Table 1:** Definitions of organ failure.

Respiratory failure	Respiratory rate ≥ 30 breaths/min; PaO_2_/FiO_2_ < 250
Haemodynamic instability	SBP < 90 mm Hg; MAP < 60 mm Hg
Neurologic impairment	Glasgow coma score < 10; Seizures
Renal impairment	Urine output < 0.5 mL/kg for last 6 hours and serum creatinine > 4 mg/dL

**Table 2:** Patient characteristics.

Characteristics	Data
Age (years)	27.3 ± 4.9
Time of admission to hospital	
Antepartum	42
Postpartum	9
Postabortal	4
Mode of admission	
Elective (booked in JIPMER)	17
Emergency (not booked in JIPMER)	38
Mode of delivery	
Vaginal delivery	10
Instrument-assisted	2
Elective caesarean section	2
Emergency caesarean section	35
Abortion/ectopic	5
Not operated	1
Referred from outside	24
Distance travelled	
<50 km	42
>50 km	13
APACHE 2 score at admission	11.8 ± 5.2

**Table 3:** Admission diagnosis.

Diagnosis	Number of patients (%)	Resp. failure	Haemo. instability	Neurologic impairment	Renal impairment
*Obstetric *					
Haemorrhage	28 (51%)	2	23	1	2
Antepartum	11/28				
Postpartum					
Post LSCS	10/28				
Post vaginal delivery	5/28				
Ruptured ectopic	2/28		2	
Hypertensive disorders				
Preeclampsia	7 (10%)	1	1		1
Eclampsia	2 (4%)		1	2	1
HELLP syndrome	2 (4%)	2	1	1	1
Sepsis	6 (11%)		4		
Anaesth. complications	1 (2%)		1		

*Nonobstetric *					
Anaemia	2 (4%)		2		
Valvular heart disease	2 (4%)	1			
Peripartum cardiomyopathy	2 (4%)	2	2		
Liver disease	1 (2%)				
Epilepsy	1 (2%)			1	
Restrictive lung disease	1 (2%)	1			

**Table 4:** Interventions undertaken in obstetric patients admitted to intensive care unit.

Interventions	Number of patients
Mechanical ventilation	47
Inotropic support	43
Arterial line insertion	34
Central venous catheter	29
Cardiac output monitoring	32
Echocardiogram	14
Ultrasound abdomen	6
CT brain	3
MRI brain	3
Renal replacement therapy	3

**Table 5:** Maternal deaths.

Case number	ICU admission diagnosis	Cause of death
1	Postpartum fulminant hepatic failure	Multiorgan failure
2	Postpartum hemorrhage	DIC, multiorgan failure
3	Antepartum hemorrhage	DIC, multiorgan failure
4	Sepsis due to intrauterine collection	Sepsis, multiorgan failure
5	Post-LSCS haemoperitoneum, hypovolemic shock	DIC, multiorgan failure
6	Postpartum hemorrhage	DIC, multiorgan failure
7	Postpartum hemorrhage	DIC, multiorgan failure
